# Subjective workload in operating room team members during robotic hernia procedures

**DOI:** 10.1007/s11701-025-02666-x

**Published:** 2025-08-21

**Authors:** Daphne Remulla, Cammy Tang, Kimberly P. Woo, William C. Bennett, Alvaro Carvalho, Brianna L. Slatnick, Kimberly S. Miles, Benjamin T. Miller, Lucas R. Beffa, David M. Krpata, Ajita S. Prabhu, Clayton C. Petro

**Affiliations:** 1 Department of Surgery, Cleveland Clinic Foundation, Cleveland, OH USA; 2 Case Western Reserve University School of Medicine, Cleveland, OH USA; 3 Cleveland Clinic Lerner College of Medicine of Case Western Reserve University School of Medicine, Cleveland, OH USA

**Keywords:** Hernia repair, Subjective workload, NASA-TLX, Circulating nurses, Scrub staff

## Abstract

While robotic surgery has been dominated by a single platform in the United States for over 25 years, the introduction of new robotic systems may have an impact on subjective workload. Therefore, we aimed to establish baseline workload measurements for operating room team members using the DaVinci surgical robot during robotic hernia procedures, providing reference points for evaluating team adaptation as new robotic platforms are introduced. Within the operating room, subjective workload refers to the physical, cognitive, and temporal demands experienced during surgical procedures. We prospectively collected NASA-TLX surveys from surgeons, circulators, and scrub staff performing robotic hernia repairs between February–December 2024. Baseline demographics and prior robotic experience were collected for each participant. Surveys assessed subjective workload across six NASA-TLX domains and surgeon assessment of case complexity relative to other procedures (Easiest 1/3, Average, Hardest 1/3). Case-specific information was extracted from the electronic medical record. We used linear mixed-effects models (LMMs) to analyze role-based and complexity-related workload, which accounts for within-subject correlation from repeated measurements collected from the same individuals across different surgical cases. A total of 131 post-operative surveys were analyzed from 14 participants across 72 robotic hernia cases. Robotic OR team members reported similar baseline workload with the highest overall workload reported by circulators (mean 30.6, 95% CI 22.1–39.2), followed by scrub staff (mean 25.1, 95% CI 16.5–33.7). Surgeons experienced the lowest overall workload (mean 24.4, 95% CI 10.2–38.6), however, demonstrated a significant stepwise increase in workload with increasing case complexity (mean 11.4 to 41.5, Cohen’s d = 3.41, 95% CI [2.31, 4.51], *p* < 0.0001), while circulators and scrub staff were unaffected. Comparisons across NASA-TLX domains showed that circulators reported significantly worse self-assessed performance (mean difference vs. surgeons: 14.97, 95% CI [8.84, 21.10], *p* = 0.001; vs. scrub staff: 9.60, 95% CI [4.21, 14.99], *p* = 0.002) and higher effort compared to other team members (mean difference vs scrub staff: 11.07, 95% CI [3.20, 18.94], *p* = 0.017). These findings provide one of the first role-specific benchmarks for intraoperative workload in robotic hernia surgery, representing essential reference metrics against which new robotic platforms can be evaluated. Further exploration of these role-specific challenges is needed to determine if there are opportunities to optimize workload to improve patient safety, team efficiency, and staff well-being as new platforms are adopted.

## Introduction

Over the past two decades, robotic-assisted surgery (RAS) has reshaped minimally invasive surgery platforms across a wide range of specialties. Most notably, for many surgical subspecialty procedures including hernia repair, robotic approaches have steadily replaced traditional laparoscopic techniques. This transformation has been largely dominated by Intuitive Surgical’s da Vinci® system, which currently represents the principal platform that surgeons, nurses, and technical staff have gained the most familiarity and expertise with since it was approved by the US Food and Drug Administration (FDA) in 2000 [1]. However, the surgical robotics landscape is poised for significant change, with multiple new platforms entering the market or nearing regulatory approval that are expected to reach widespread clinical adoption in the coming years [2, 3]. The introduction of these novel technologies will likely impose additional cognitive and physical demands on entire surgical teams as they adapt to new interfaces, workflows, and technical requirements.

Hernia repair procedures offer a particularly compelling context for exploring workload in robotic surgery, as it encompasses a wide spectrum of complexity, ranging from straightforward primary ventral hernia repairs to complex abdominal wall reconstructions, all of which may contribute to increased workload for the entire surgical team. Despite these demands, the impact of robotic hernia repair on non-surgeon team members remains largely unexplored [4–6].

This study aims to establish baseline subjective workload measures experienced by various members of the operating room (OR) team during robotic hernia procedures. Using the National Aeronautics and Space Administration-Task Load Index (NASA-TLX) as a subjective workload assessment tool, we investigate current workload patterns across roles to create reference points that can be used to evaluate adaptation to emerging robotic platforms in the future. Additionally, we aim to explore factors that contribute to workload in these team members to better understand the determinants of intraoperative subjective workload burden across the surgical team.

## Methods

This study utilized a retrospective analysis of prospectively collected data from an Institutional Review Board approved, single institution registry dedicated to workload in operating room team members in robotic surgery. We examined surveys completed between February 14, 2024, and December 31, 2024, focusing exclusively on robotic hernia procedures. The study population comprised surgeons, circulating nurses, and scrub staff who function independently in the robotic operating room setting and participate in robotic hernia procedures. Scrub staff are comprised of scrub nurses and scrub technicians. At our institution, hernia procedures are performed by general surgeons with fellowship training in advanced abdominal wall reconstruction as a part of our Center for Abdominal Core Health. Circulating nurses and scrub staff within our department participate in all general surgery procedures, and only those trained in robotic procedures are assigned to robotic general surgery cases. All participants were included in the registry after providing written informed consent.

Participant data were collected using standardized survey instruments at two timepoints: (1) baseline questionnaire upon enrollment capturing demographic information and prior robotic surgery experience, and (2) post-operative surveys administered immediately at the conclusion of each robotic procedure. These surveys documented subjective workload using the National Aeronautics and Space Administration-Task Load Index (NASA-TLX), a validated multi-dimensional scale designed to assess operator workload in complex environments [7]. The NASA-TLX evaluates six domains: mental demand, physical demand, temporal demand, performance, effort, and frustration, each scored on a scale of 0–100. Higher scores indicate greater perceived workload except for performance, where higher scores represent worse self-assessed performance. Additionally, participants reported their operative role, same-day case load, and surgeon assessment of case complexity relative to other procedures (easiest 1/3, average, hardest 1/3). Case-specific information was extracted from the electronic medical record (Epic), including robotic platform, procedure type, and operative time. All data were securely stored in REDCap® (Research Electronic Data Capture), a secure network and firewall-protected electronic database.

### Statistical analysis

Descriptive statistics were used to summarize participant characteristics, procedure details, and survey responses. Categorical variables were reported as frequencies and percentages, while continuous variables were presented as means with standard deviations or medians with interquartile ranges based on distribution normality.

To establish reliable baseline overall workload measures for each operating room team role (primary surgeon, circulator, and scrub staff), we calculated the unweighted mean of six NASA-TLX subscales: mental, physical, temporal, performance, effort, and frustration. We used unweighted NASA-TLX scores for analysis, consistent with prior studies showing that weighted and unweighted scores are highly correlated and yield comparable results [8, 9].

Because simple descriptive statistics do not account for the hierarchical structure of our data, we used a linear mixed-effects model. This approach provides adjusted estimates of workload scores that properly account for multiple observations per participant as well as unequal observations per group. These adjusted estimates offer more accurate baseline references by accounting for individual participants' baseline differences while still capturing overall patterns across all participant responses .

In addition to establishing these baseline workload measurements, we conducted exploratory analyses to examine differences in perceived workload across roles and investigate factors influencing workload. We used a mixed-effects model approach, thereby still accounting for repeated measures from the same individuals, and included two factors in the model: (1) the surgical team role (surgeon, circulator, or scrub staff), and (2) the complexity of the procedure, as assigned by the surgeon. The rationale for using the surgeon-assigned complexity rating is multifold. One, the surgeon’s assessment best reflects the full technical and cognitive demands of an operation, including anatomical difficulty, procedural nuances, and intraoperative decision-making. Second, using a single, consistent perspective for assigning case complexity provides a more standardized comparison and allows us to explore whether greater surgical complexity translates into increased workload across the entire team. Using this model, we compared adjusted workload scores between roles and within NASA-TLX subscales.

All statistical analyses were performed using R version 4.2.0 (R Foundation for Statistical Computing, Vienna, Austria) with statistical significance set at *p* < 0.05.

## Results

**Fig. 1:**
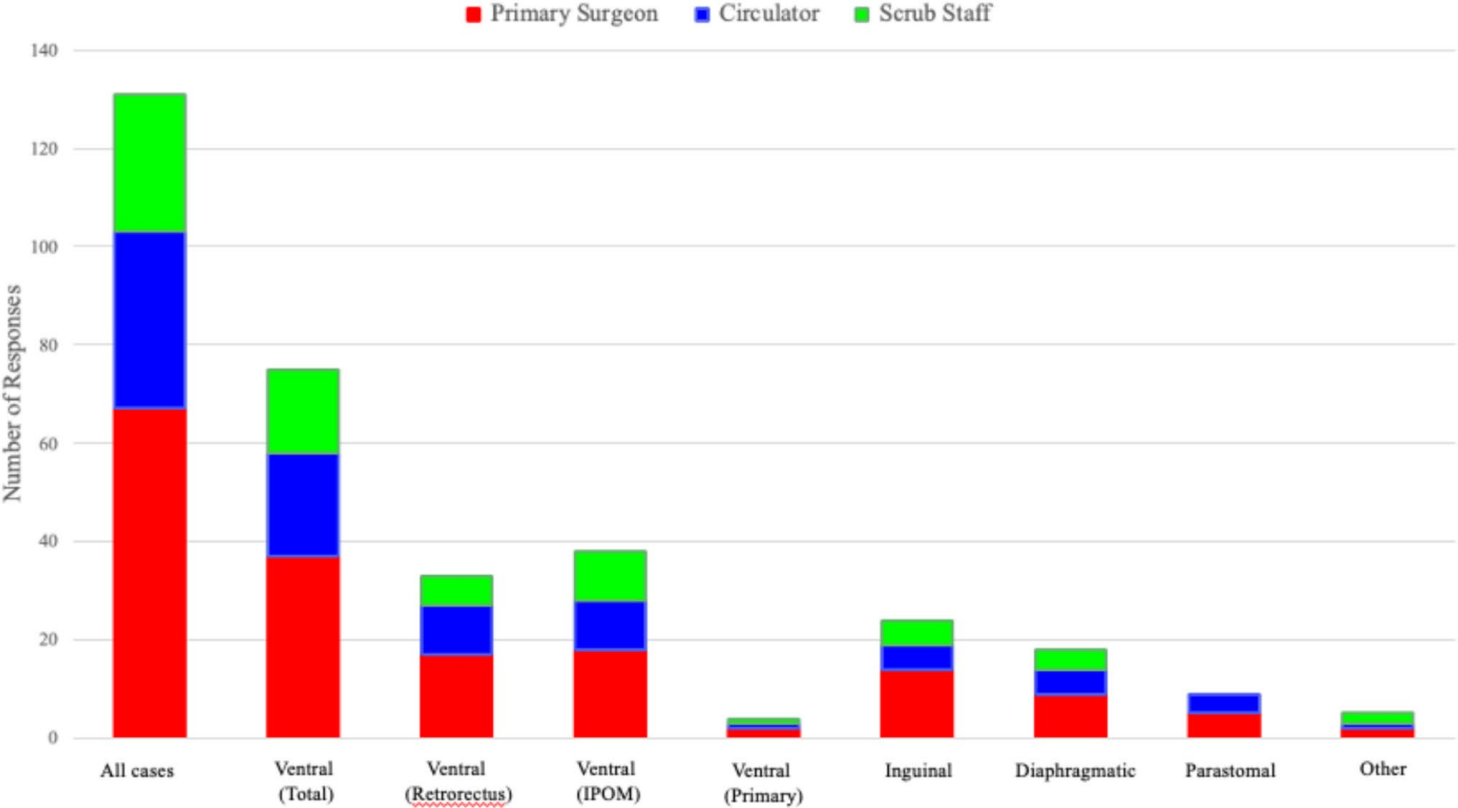
Survey Responses by Hernia Procedure and Operating Room Team Role. This stacked bar chart shows the number of NASA-TLX responses across all cases and by specific hernia case types, stratified by operating room role. *IPOM – Intraperitoneal Onlay Mesh

Participant demographics are detailed in Table 1. A total of 14 participants were enrolled, including three surgeons, six circulating nurses and five scrub staff. All primary surgeons were male, while all circulators and scrub staff were female. All participants had prior robotic surgical experience, with primary surgeons reporting the highest number of previous robotic cases (median 175, IQR 100–500) and months of robotic experience (median 65 months, IQR 50–96). Our analysis encompassed 131 completed surveys, with primary surgeons accounting for 51.1% (67) of the total survey responses, circulators 27.5% (36), and scrub staff 21.4% (28) (Fig. 1).

**Table 1:** Participant Demographics

Characteristic	ALL (n = 14)	Surgeon, (N = 3)	Circulator, (N = 6)	Scrub Staff, (N = 5)
Age, median (IQR)	36.5 (31–41)	40 (36–45)	35 (31–38)	36 (30–41)
Gender, N (%)				
Female	11 (78.6%)	0 (0.0%)	6 (100.0%)	8 (100.0%)
Male	3 (21.4%)	3 (100.0%)	0 (0.0%)	0 (0.0%)
Race, N (%)				
White	13 (92.9%)	3 (100%)	5 (83.3%)	5 (100%)
More than one race	1 (7.1%)	0 (0.0%)	1 (100%)	0 (0.0%)
Baseline Robotic Cases, median (IQR)	44 (30–100)	175 (100–500)	45 (30–100)	30 (12.5–44)
Baseline Robotic Experience, median (IQR)	36 (15–60)	65 (50–96)	24 (15–48)	30 (10–60)

**Fig. 2:**
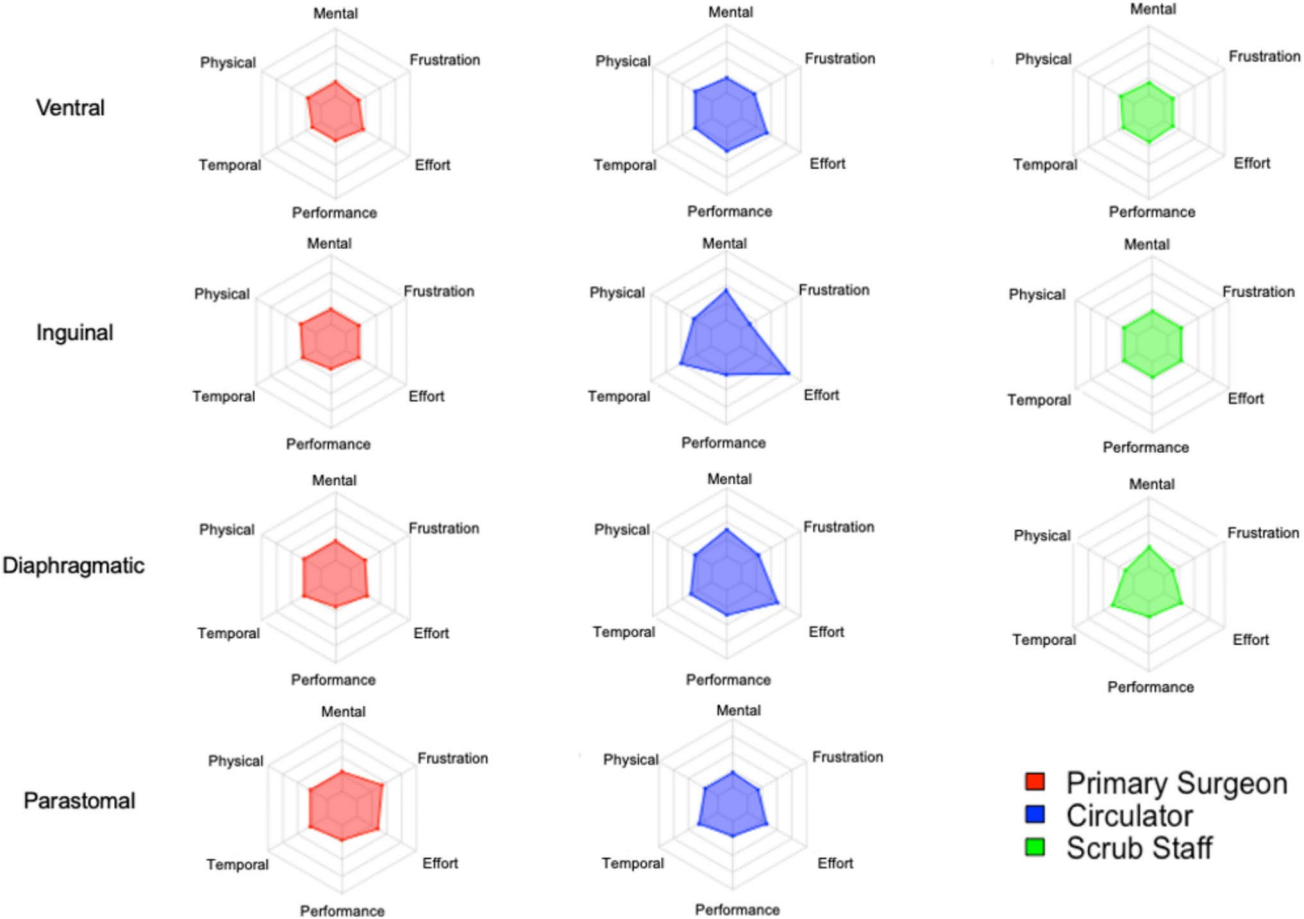
NASA-TLX Domains by Hernia Procedure and Operating Team Role. Radar plots illustrate mean scores across the six NASA-TLX subdomains—mental demand, physical demand, temporal demand, performance, effort, and frustration—stratified by surgical role (primary surgeon, circulator, scrub staff) and case type (ventral, inguinal, diaphragmatic, parastomal). Each plot represents average subjective workload ratings for that role–case combination. Higher values indicate greater perceived workload in each domain

Procedure details are outlined in Table 2. Ventral hernias constituted the majority of cases (n = 41, 55.6%), with an approximately equal distribution between intraperitoneal onlay mesh (IPOM) procedures (n = 20, 52.6%) and retrorectus repairs (n = 18, 47.4%). Inguinal hernias (n = 15, 20.8%) were the next most common, followed by diaphragmatic (n = 8, 11.1%) and parastomal hernias (n = 6, 8.3%). Diaphragmatic repairs included hiatal hernias, paraesophageal hernias and diaphragmatic hernias. Parastomal hernia repairs were performed using an intraperitoneal onlay mesh Sugarbaker technique. Four procedures (5.6%) were categorized as “Other,” which included mesh excision or diagnostic procedures for chronic abdominal pain in patients with previous hernia repair. Retrorectus repairs had the longest median operating time (220.5 min, IQR 194–276), followed by parastomal hernia repairs (165.5 min, IQR 128–193). The highest overall subjective workload was observed in circulators during inguinal cases (median 29.2, IQR 27.5–30), followed by circulators during diaphragmatic hernia repairs (median 27.1, IQR 13.8–40.8). The lowest workload was reported by scrub staff in “Other” procedures (median 5, IQR 5–8.3) and by surgeons during retrorectus repairs (median 6.7, IQR 5–15.8) (Table 2). Figure 2 displays NASA-TLX scores across each domain for different operating room team roles during the observed hernia procedures.

**Table 2:** Hernia Procedures – Case Characteristics and Median NASA-TLX Score by Role

Procedure	N (%)	Operative Time (min)	Overall NASA-TLX, median (IQR)		
			Surgeon	Circulator	Scrub Staff
Total Ventral	41 (55.6%)	150 (105–220)	15.5 (5–73)	22 (12–57)	16.5 (5–53)
Ventral—IPOM*	20 (48.8%)	221 (194–276)	6.7 (5–16)	21.3 (19–26)	14.2 (10–18)
Ventral—Retrorectus	19 (46.3%)	107.5 (89.5–144)	15.8 (13–27)	20.9 (15–45)	15.4 (11–42)
Ventral—Primary	2 (4.9%)	112 (50–174)	12.5 (10–15)	15 (15–15)	5 (5–5)
Inguinal	15 (20.8%)	120 (100–143)	16.5 (5–64)	29 (22–56)	15 (6–43)
Diaphragmatic	9 (12.5%)	145 (141–239)	15 (5–57)	20 (10–46)	22.5 (16–29)
Parastomal	6 (8.3%)	165.5 (117–200)	13.5 (5–58)	21 (11–58)	N/A
Other	2 (2.9%)	62 (40–84)	7.5 (5–10)	15 (15–15)	8.5 (5–12)

*IPOM – Intraperitoneal Onlay Mesh

**Fig. 3:**
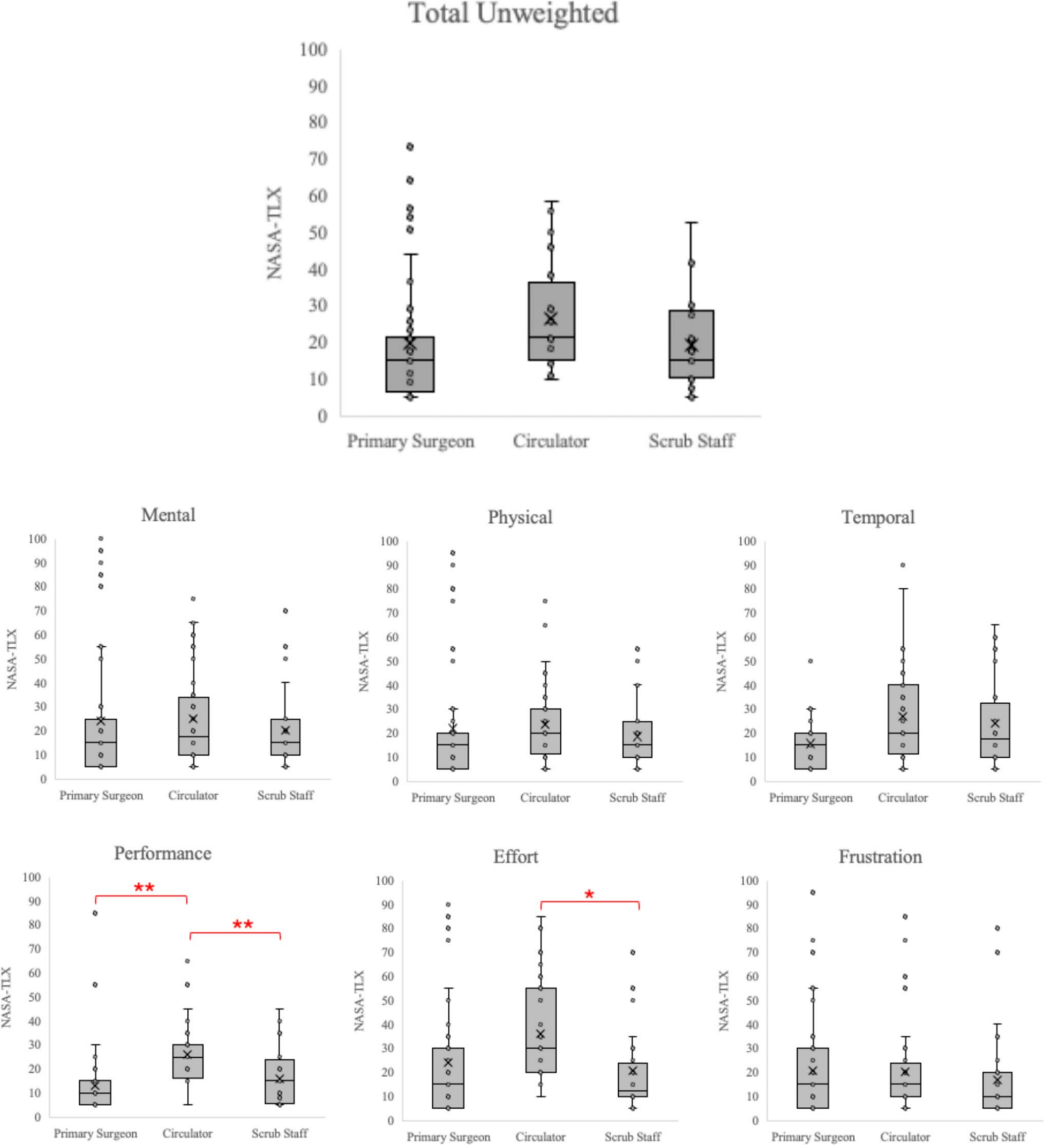
Overall NASA-TLX by Operating Room Team Role and Case Complexity. Box plots showing the distribution of NASA-TLX subdomain scores across operating room team roles for all robotic hernia procedures. Each box represents the interquartile range (25th-75th percentile) with median values indicated by the horizontal line within each box. The "X" marks represent mean values, and whiskers extend to 1.5 times the interquartile range. Higher scores indicate greater perceived workload in all domains except performance, where higher scores represent worse self-assessed performance. Asterisks indicate statistically significant differences between groups (*p < 0.05, **p < 0.01) based on mixed-effects modeling accounting for repeated measures (participant ID), role and case complexity

Unweighted NASA-TLX and domain scores stratified by operating room team role are presented in Fig. 3. Circulators reported the highest overall workload (mean 30.6, 95% CI 22.1–39.2), followed by scrub staff (mean 25.1, 95% CI 16.5–33.7), with surgeons experiencing the lowest overall workload (mean 24.4, 95% CI 10.2–38.6). However, these differences were not statistically significant (*p* = 0.64).

**Fig. 4:**
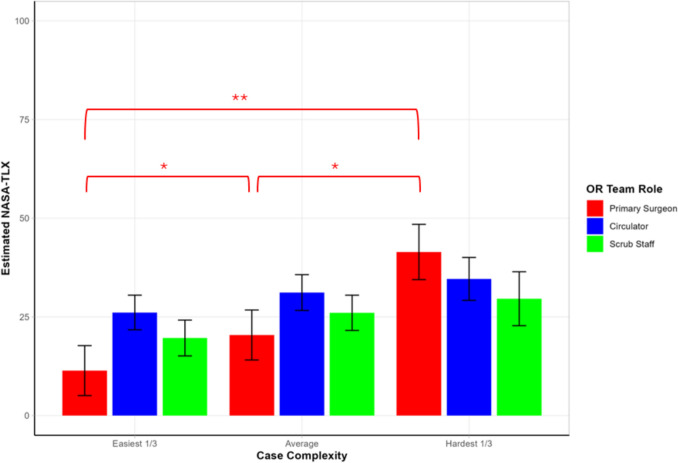
Pairwise Comparisons of Case Complexity By Role. Bar chart displaying estimated NASA-TLX overall workload scores stratified by operating room team role across three levels of surgeon-assigned case complexity (easiest 1/3, average, hardest 1/3). Error bars represent the standard error of the mean. Red brackets with asterisks indicate statistically significant pairwise comparisons for primary surgeons across complexity levels (*p < 0.005, **p < 0.0001). Results are derived from mixed-effects modeling accounting for repeated measures (participant ID), role, and case complexity. Primary surgeons demonstrated a significant stepwise increase in workload with increasing case complexity, while circulators and scrub staff workload remained unaffected by complexity changes

For primary surgeons, there was a clear stepwise increase in workload with increasing surgical case complexity, with adjusted NASA-TLX scores increasing from 11.4 (SE = 5.28) in the lowest third of case complexity to 20.4 (SE = 5.29) in average complexity cases, and 41.6 (SE = 6.03) in the hardest 1/3. All pairwise contrasts were statistically significant: easiest 1/3 vs average; Cohen’s d = 0.83, 95% CI [0.30, 1.36], *p* = 0.001), easiest vs hardest 1/3 (Cohen’s d = 3.41, 95% CI [2.31, 4.51], *p* < 0.0001), and average vs hardest 1/3 (Cohen’s d = 2.11, 95% CI [1.20, 3.02], *p* < 0.0001), indicating substantial perceived workload increases for surgeons with increasing complexity. For circulators and scrub staff, there was no difference in workload as surgeon-assigned case complexity increased (Fig. 4).

Within workload subscales, circulators perceived significantly worse self-assessed performance (mean difference vs. surgeons: 14.97, 95% CI [8.84, 21.10], *p* = 0.001; vs. scrub staff: 9.60, 95% CI [4.21, 14.99], *p* = 0.002) and higher effort compared to other team members (mean difference vs scrub staff: 11.07, 95% CI [3.20, 18.94], *p* = 0.017). Mental demand, physical demand, temporal demand, and frustration subscale scores were similar across all roles (Fig. 3).

## Discussion

In this single-institution study of robotic hernia procedures, we established baseline workload measures across different operating room team roles during robotic hernia repairs. Our findings provide an initial framework and methodology for evaluating workload across OR team members, though larger multi-institutional studies with more participants are needed to establish robust benchmarks for clinical practice. While our exploratory analysis found that circulating nurses reported significantly worse self-assessed performance and higher effort scores during robotic hernia procedures, with case complexity serving as the primary driver of perceived workload rather than team role, the primary contribution of this work is as one of the first role-specific, real-world benchmarks for intraoperative workload in robotic hernia surgery.

Robotic-assisted surgery has largely relied on the DaVinci surgical robot as the single dominant platform, however, with multiple new systems entering the market, surgical teams will soon face new cognitive and physical demands. Indeed, a recent overview of emerging multi-visceral robotic platforms by the Society of American Gastrointestinal Surgeons (SAGES) revealed a growing diversity of systems—ranging from single-arm to multi-arm designs with varying footprints to optimize OR space, integrated AI for automated camera control, and telesurgery capabilities across thousands of kilometers [2]. This rapid expansion underscores a fundamental shift toward more versatile, cost-effective, and globally accessible solutions, with several new entrants targeting lower price points or more user-friendly interfaces to ease the transition for experienced surgeons.

In this evolving backdrop, our baseline workload data provide an important reference point for evaluating how these changes impact the entire OR team. While previous studies have evaluated surgeon workload alone in robotic compared to laparoscopic IPOM [10], our study expands this focus to include the entire surgical team. By establishing baseline workload measures across roles and complexity levels in hernia surgery, we offer both a reference point and framework for evaluating how future innovations may alter the cognitive and physical demands placed on the broader surgical team [2, 3, 11–14]. This approach acknowledges that successful implementation of new surgical technologies depends not only on the surgeon’s adaptation but on the entire team’s ability to integrate these tools into their workflow.

Importantly, while existing studies assess surgeon workload across platforms, most treat workload as static and ignore the repeated, team-based nature of intraoperative data [4, 5, 11, 15–17]. Our use of mixed-effects modeling to account for repeated measures and within-subject correlation represents a methodological strength, revealing that over half of the variance in workload was attributable to individual-level differences. This approach offers a more accurate means of generating reference values and tracking adaptation to new systems over time. As survey-based workload assessments remain common in perioperative research, future workload studies should incorporate mixed-effects modeling or other strategies to account for repeated measures and intra-individual variability. It is also worth noting that while newer tools such as SURG-TLX exist [18], we selected the NASA-TLX due to its robust and widespread validation, extensive use in surgical workload research, and strong alignment with core domains relevant to robotic surgery, including mental and physical demand [19, 20]. While SURG-TLX offers surgery-specific adaptations, NASA-TLX enables broader comparability with existing literature across roles, as it more commonly used in both surgical and nursing workload research [21].

Though we found no statistically significant difference in overall workload between team roles, suggesting that perceived workload is broadly comparable across team members when averaged across cases, we observed a trend of higher perceived workload for non-surgeons, particularly circulating nurses. On further exploratory analysis, circulators reported significantly worse self-assessed performance and higher effort scores compared to other team members. Furthermore, while our finding that surgeon-reported workload increases with surgeon-assigned case complexity is expected, the notion that circulators and scrub staff are unaffected by increasing surgeon-perceived case complexity is another noteworthy exploratory finding. This suggests that the additional burden of complex procedures is not evenly distributed across the operating room team. Surgeons may absorb a disproportionate share of the overall workload as case complexity increases, reflecting the additional cognitive and technical demands they face in more challenging procedures. In contrast, supporting team members may be more impacted by task execution demands and perceived expectations for maintaining efficiency and accuracy. These role-specific workload patterns highlight the need for targeted strategies to support each team member according to the unique demands of their role.

These differences are consistent with prior studies, reporting variability in intraoperative workload across surgical roles [15, 16, 22]. This dissociation between surgeon-assigned case complexity and perceived workload in supporting roles raises important questions about how workload is experienced and managed across team members and warrants further study. Robotic platform design has been found to meaningfully influence intraoperative workload across surgical team members, reinforcing the need for structured, role-specific workload data such as that provided in the present study [11, 23].

Quantifying workload in the operating room is important as limited studies suggest that greater workload may be implicated in patient safety outcomes, with increased patient safety incidents and diminished attention sensitivity associated with higher workload demands in nursing staff [24, 25]. Our observed differences in workload, particularly higher effort and lower performance scores among circulators and greater surgeon workload with increasing case complexity, may indicate areas where targeted training or interventions might be beneficial, particularly as new platforms with varied instrument designs and console layouts are introduced. For example, role-specific training modules that address unique workload challenges, coupled with team-based simulation exercises focused on workflow optimization and distributing workload more evenly across complex cases, may help enhance team performance and patient safety as the adoption of new surgical robotics platforms introduces additional workload demands.

However, several important limitations must be acknowledged. First, this was a single-institution study with a relatively small sample size, which precludes establishing definitive benchmarks and may not be representative of workload patterns in other institutions or practice settings. Second, our study has a substantial risk of confounding between surgical role, gender, and participant experience level. Our study cohort revealed distinct gender distribution patterns across surgical team roles, with all surgeons being male and all circulating and scrub staff being female. This demographic pattern makes it impossible to distinguish whether observed workload differences reflect role-specific demands, gender-related factors, or some combination thereof. Moreover, the robotic experience disparity between roles (surgeons with a median 65 months vs. circulators with 24 months vs. scrub staff with 30 months) further introduces the possibility that observed differences reflect learning curve effects rather than inherent role-based demands. We deliberately recruited experienced surgeons to establish baseline workload measures for surgeons who had plateaued in their comfort with the da Vinci platform. This methodological choice enabled us to isolate surgeon-assigned case complexity as the primary variable without introducing surgeon learning curve effects as an additional confounder; however, this approach likely contributed to the observed role-based workload differences. Had we included less experienced surgeons, who would likely experience greater workload demands, this may have reduced the workload gap between surgeons and other team members and altered the relationship between case complexity and surgeon workload through steeper increases or different patterns altogether. Without a more balanced sample across gender and experience levels for each role, it is unclear whether the lack of association between nursing staff workload and surgeon-assigned complexity is truly role-specific or reflects the current experience distribution. This fundamental confounding limits the generalizability of our role-specific findings and necessitates cautious interpretation of our results. Third, our dataset had limited representation of high-complexity cases, which may have overestimated the effect of complexity on workload, given that even a small number of outlier cases can disproportionately influence model estimates in small samples. Fourth, while we relied on the surgeon’s rating of case complexity as a reference point, this inherently reflects one perspective and may not capture dimensions of complexity that affect other team members differently, such as coordination challenges or environmental stressors. Our finding that non-surgeon team members demonstrated no change in workload with increasing surgeon-perceived complexity may reflect the limitations of this surgeon-centric complexity assignment method rather than the true absence of complexity-related workload demands for these roles. Future studies should consider role-specific complexity measures or objective indicators that better capture the multidimensional nature of case difficulty across all team members. Additionally, the NASA-TLX instrument lacks an established minimal clinically important difference, which limits our ability to determine the clinical significance of observed workload variations between roles and case complexities. Finally, these analyses were exploratory and not pre-specified as primary endpoints. Although mixed-effects modeling is appropriate for repeated measures and hierarchical data, our interpretation of workload differences across roles and subdomains should be viewed as hypothesis-generating rather than definitive. Further research with larger, more diverse samples and prospective validation is needed to confirm these patterns and better understand the team-wide impact of surgical complexity on workload.

## Conclusion

In conclusion, our study provides workload measurements across different team roles during robotic hernia procedures using the currently dominant robotic platform. These findings establish reference points that can be used to evaluate adaptation to emerging robotic platforms as they enter clinical practice. Future multi-institutional studies with larger, more diverse samples and balanced gender representation across roles are needed to refine these baseline measures and strengthen their utility for evaluating new surgical technologies. By understanding the workload impact of technological transitions on the entire surgical team, we can better support successful implementation of innovative robotic platforms that aim to improve surgical outcomes.

## Data Availability

The data that support the findings of this study are not openly available due to reasons of participant confidentiality and the sensitivity of employee data.
